# Prevalence of *Strongyloides* in Southeast Asia: a systematic review and meta-analysis with implications for public health and sustainable control strategies

**DOI:** 10.1186/s40249-023-01138-4

**Published:** 2023-09-13

**Authors:** Abigail Hui En Chan, Teera Kusolsuk, Dorn Watthanakulpanich, Wallop Pakdee, Pham Ngoc Doanh, Azlin Mohd Yasin, Paron Dekumyoy, Urusa Thaenkham

**Affiliations:** 1 Department of Helminthology, Faculty of Tropical Medicine, Mahidol University, Bangkok, Thailand; 2 Institute of Ecology and Biological Resources, Graduate University of Science and Technology, Vietnam Academy of Science and Technology, Hanoi, Vietnam; 3 Department of Parasitology and Medical Entomology, Faculty of Medicine, Universiti Kebangsaan Malaysia, Selangor, Malaysia

**Keywords:** *Strongyloides*, Strongyloidiasis, Southeast Asia, Prevalence, Systematic review

## Abstract

**Background:**

Strongyloidiasis, caused by the nematodes *Strongyloides stercoralis* and *Strongyloides fuelleborni*, is estimated to affect over 600 million individuals worldwide. The disease is endemic in Southeast Asia, where a warm-humid climate and socio-economic conditions maintain the parasite’s life cycle and transmission. However, the current diagnostic methods may not be sufficiently sensitive, suggesting that the true prevalence of strongyloidiasis could be seriously underestimated in this. This study aims to determine the prevalence of strongyloidiasis in Southeast Asia through a systematic review and meta-analysis and to discuss the implications of the estimated prevalence on diagnostic approaches and control strategies.

**Methods:**

Following PRISMA guidelines, we conducted a systematic literature search in PubMed and Google Scholar databases to identify studies reporting *Strongyloides* prevalence data in the 11 Southeast Asian countries up to December 2022. A random effects model was employed to estimate the pooled prevalence of *S. stercoralis* at both regional and country levels.

**Results:**

Out of 3722 articles identified, 224 met our inclusion criteria. For *S. stercoralis* specifically, we found 187 articles, of which 52.4% were from Thailand. All Southeast Asian countries, except Brunei, had at least one study on *Strongyloides* prevalence. The estimated pooled prevalence of *S. stercoralis* regionally was 12.7% (95% *CI* 10.70–14.80%), ranging from 0.4 to 24.9% at the country level. Cambodia had the highest pooled prevalence (24.9%, 95% *CI* 15.65–35.38%), followed by Lao PDR (16.5%, 95% *CI* 9.50–24.95%). Moreover, we obtained a pooled prevalence of 10% (95% *CI* 7.06–13.52%) in a group comprising immigrants, workers, and veterans from Southeast Asian countries. *S. stercoralis* infects various host types, including nonhuman primates, domestic dogs and cats, rodents, and transport carriers such as cockroaches and vegetables.

**Conclusions:**

A high prevalence of strongyloidiasis in Southeast Asia was revealed, highlighting the importance of the region’s ongoing research, surveillance, and control efforts. Factors contributing to the strongyloidiasis transmission include the role of animal hosts, the impact of global connectivity, and the significance of the co-endemicity of other *Strongyloides* species. Based on these findings, a multi-pronged One-Health approach is essential for sustainable intervention and control.

**Supplementary Information:**

The online version contains supplementary material available at 10.1186/s40249-023-01138-4.

## Background

Strongyloidiasis is a neglected tropical disease that mainly affects humans in tropical and subtropical regions, with an estimated global prevalence of over 600 million people [1]. The disease is caused by the nematodes *Strongyloides stercoralis* and *Strongyloides fuelleborni* [2]. Clinical manifestation of *S. stercoralis* infection in human can range from asymptomatic to chronic, depending on the infection intensity. The parasite can live in human body for many cycles if left untreated through autoinfection, where larval infectivity can be achieved without leaving the host [3, 4]. Immunocompromised individuals are particularly susceptible to hyperinfection, leading to mortality [5, 6]. On the other hand, *S. fuelleborni* primarily infects various nonhuman primates, and although cases of human infection have been reported in African and Southeast Asian countries, autoinfection is unlikely as eggs are excreted in feces and hatch in the external environment [4, 7–10].

Despite the medical significance of strongyloidiasis, there is a consensus that the global prevalence was underestimated due to the low sensitivity of current diagnostic techniques. Currently, the optimal parasitological method for *S. stercoralis* diagnosis is the detection of larvae in fecal samples [3, 11] using methods such as the Baermann-Mores, agar plate culture (APC), and Harada-Mori culture. However, other parasitological techniques unsuitable for detecting larvae but more suited for detecting eggs in fecal samples are still widely used due to their ease of application. These parasitological techniques can lead to underestimating light infections when larval output is intermittent and low [12].

Southeast Asian region comprises 11 countries, collectively inhabiting more than 600 million people [13]. The countries include Thailand, Cambodia, Lao People’s Democratic Republic (Lao PDR), Myanmar, Vietnam, Indonesia, Malaysia, The Philippines, Singapore, Timor-Leste, and Brunei. Southeast Asia is rapidly emerging as a global economic hub for technology and innovation, enhancing its popularity as an ideal destination [14, 15]. Furthermore, an estimated 23.6 million Southeast Asian migrants reside outside their country of origin, with labor migration being the dominant trend in recent decades [16]. With increased global connectivity, patterns of human migration, and favorable climatic, ecological, and socio-economic conditions, Southeast Asia is an ideal environment for transmitting strongyloidiasis. The World Health Organization (WHO) has recently included strongyloidiasis as a soil-transmitted helminth (STH) targeted for control, emphasizing its medical importance and the risk of transmission [17].

This study aims to estimate the prevalence of *S. stercoralis* infection in 11 Southeast Asian countries using a systematic review and meta-analysis and to discuss the implications of the estimated prevlance for diagnosis and control strategies. In view of the WHO’s inclusion of *S. stercoralis* as another STH targeted for control in 2030, the results can provide valuable data for strongyloidiasis control strategies in Southeast Asia.

## Methods

### Search strategy

The systematic review and meta-analysis followed the PRISMA guidelines (http://www.prisma-statment.org/) (Additional file 1). In December 2022, identification of all publications regarding *Strongyloides* in Southeast Asia was searched in PubMed and Google Scholar databases. The keywords used were *Strongyloides* AND the 11 countries in Southeast Asia, namely, Thailand, Laos, Cambodia, Myanmar, Vietnam, Timor-Leste, Indonesia, Malaysia, Brunei, Philippines, Singapore. Two researchers performed the data search in the databases, and the titles and abstracts were evaluated independently.

### Exclusion and inclusion criteria

The articles from the search were input into a Microsoft Office Excel (Microsoft Corporation, Albuquerque, USA) spreadsheet, and duplicates were removed. Articles were eligible for inclusion if they met the following criteria: (1) data showing the prevalence of *Strongyloides* from any of the countries in Southeast Asia regardless of host species, (2) articles related to immigrants, workers, veterans (IWV) in Southeast Asian countries, (3) peer-reviewed articles containing original data, and (4) articles with full-text access with abstract. Articles not meeting the above criteria were excluded, such as review articles, case reports, those with no full-text and abstract, and those not in English (excluding Thai). In case of discrepancies regarding the inclusion or exclusion of a study, the team discussed the matter to reach a consensus agreement.

### Data extraction

The articles were grouped according to each country and a group containing articles from IWV (all from Southeast Asia). The data that was retrieved from each included article were: first author name, year of publication, country and province or state of study, study population, *Strongyloides* species, host, a diagnostic method used, total sample size, positive samples, and prevalence (Additional files 2 and 3). If the number of positive samples or prevalence were not calculated, either value was inferred from the total sample size and prevalence or the number of positive samples. If more than one diagnostic method was used, the method with the highest prevalence was selected for meta-analysis to ensure that strongyloidiasis was not underestimated in this meta-analysis.

### Statistical approach and meta-analysis

Meta-analysis was conducted for *S. stercoralis*, and the overall pooled prevalence was calculated with 95% confidence intervals (*CI*). A sub-analysis was performed according to the year of publication for the overall pooled prevalence, where the time periods selected were: year 1970 to 2000 (before millennium) and 2001 to 2022 (after millennium). The dataset from Thailand was also analysed based on these two time periods due to the high number of studies available. Another subgroup analysis was performed to calculate pooled prevalence per country. The pooled prevalence estimates were calculated using the alpha method for the random effects model based on the inverse variance method for measuring weight. Cochran Q test and the inconsistency index (*I*^*2*^) were used to assess the degree of heterogeneity among studies, with values of more than 75% considered high heterogeneity. Publication bias was calculated using Egger’s regression and Begg’s test, where a *P*-value of < 0.05 indicated the presence of publication bias.

The calculated pooled prevalence per country was plotted on a map, with the degree of prevalence indicated by the color intensity. Additionally, the diagnostic methods used for each country were summarized as percentages and plotted on the map. All statistical, meta-analysis, and map generation were conducted in R studio version 1.4.1717 (RStudio, PBC, Boston, USA) [18].

In addition to calculating pooled prevalence estimates for each country, regional prevalence rates of *S. stercoralis* were determined for Thailand, Cambodia, Lao PDR, and Malaysia by averaging the prevalence data from the relevant studies. These countries were selected due to their high number of *S. stercoralis* prevalence studies. The regions were as follows: Thailand was divided into North, Northeast, Central, South; Cambodia into Northwestern, Elephant Mountains and Cardamom, Mekong lowland and Eastern; Lao PDR into North, Central and South; and Malaysia into Peninsular and East.

The proportions of the types of diagnostic tests used to detect *S. stercoralis* were also calculated and summarized, with tests falling into one of four categories: parasitological only, serological only, molecular only, and a combination of any two methods (which includes any pairings of the aforementioned methods).

## Results

### Data characteristics

**Fig. 1:**
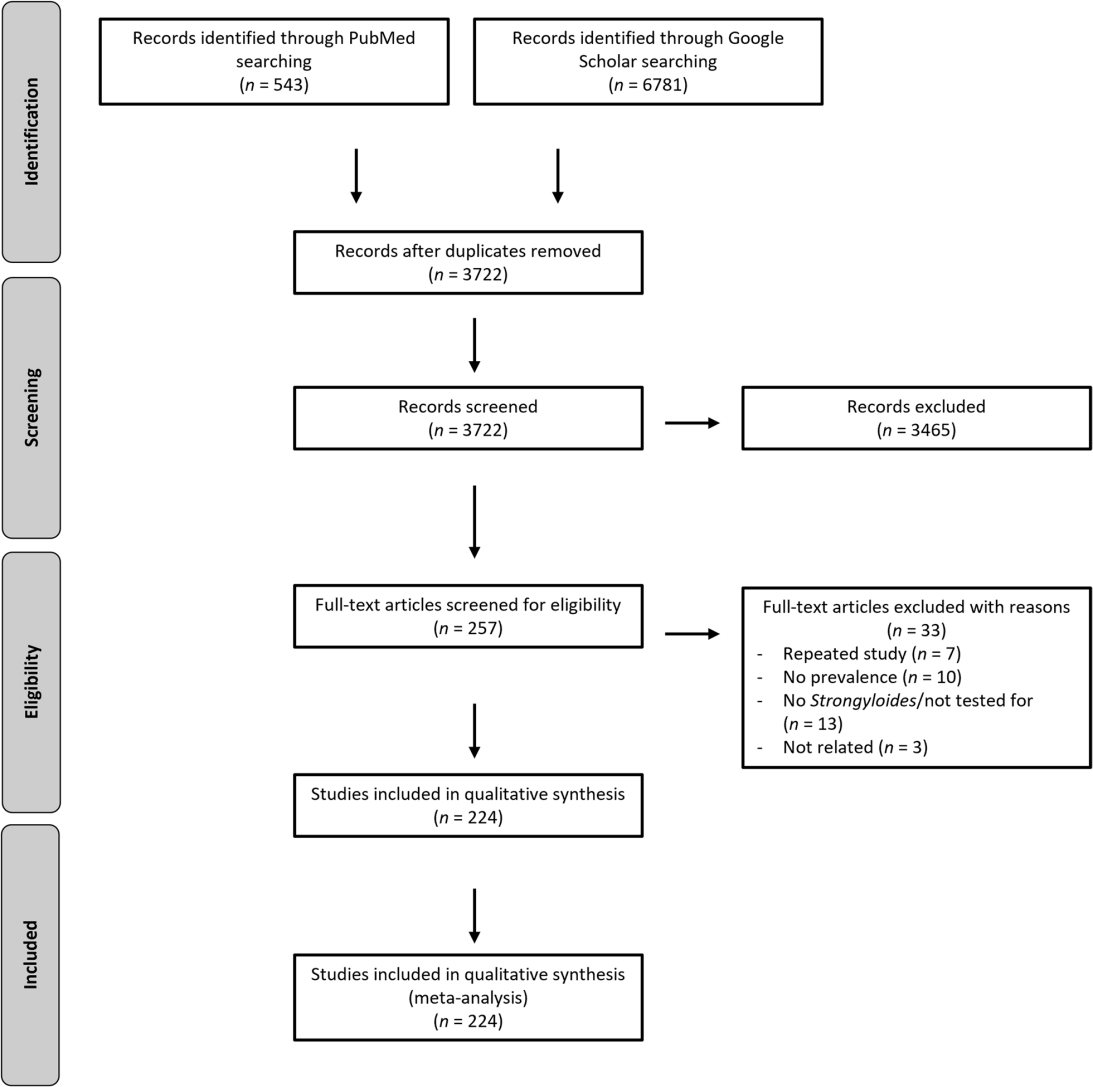
Overview of search strategy conducted for the systematic review

A total of 543 and 6781 records were found in PubMed and Google Scholar databases, respectively. From both databases, 3722 publications were obtained after duplicates were removed. Of these, 257 publications were screened for their eligibility. Thirty-three articles were excluded, of which seven studies presented repeat data, 10 had no record of prevalence data, 13 did not test for *Strongyloides*, and three were unrelated. Finally, 224 publications were included in qualitative synthesis and meta-analysis (Additional files 2 and 3). Figure 1 presents the flow diagram representing the articles selection process.

Of the 11 countries in Southeast Asia, records were found for 10 of them: Thailand, Cambodia, Lao PDR, Myanmar, Vietnam, Indonesia, Malaysia, The Philippines, Singapore, and Timor-Leste. No reports on *Strongyloides* prevalence were found from Brunei. Of the 224 publications, the top three were obtained from Thailand (49.5%), followed by the IWV group (10.4%) and Malaysia (9.6%). Specifically, for *S. stercoralis*, 187 publications were obtained, with most studies (52.4%) conducted in Thailand. Moreover, a significant proportion of studies (12.6%) were observed for the IWV group.

### *Strongyloides stercoralis* prevalence in Southeast Asia

**Fig. 2:**
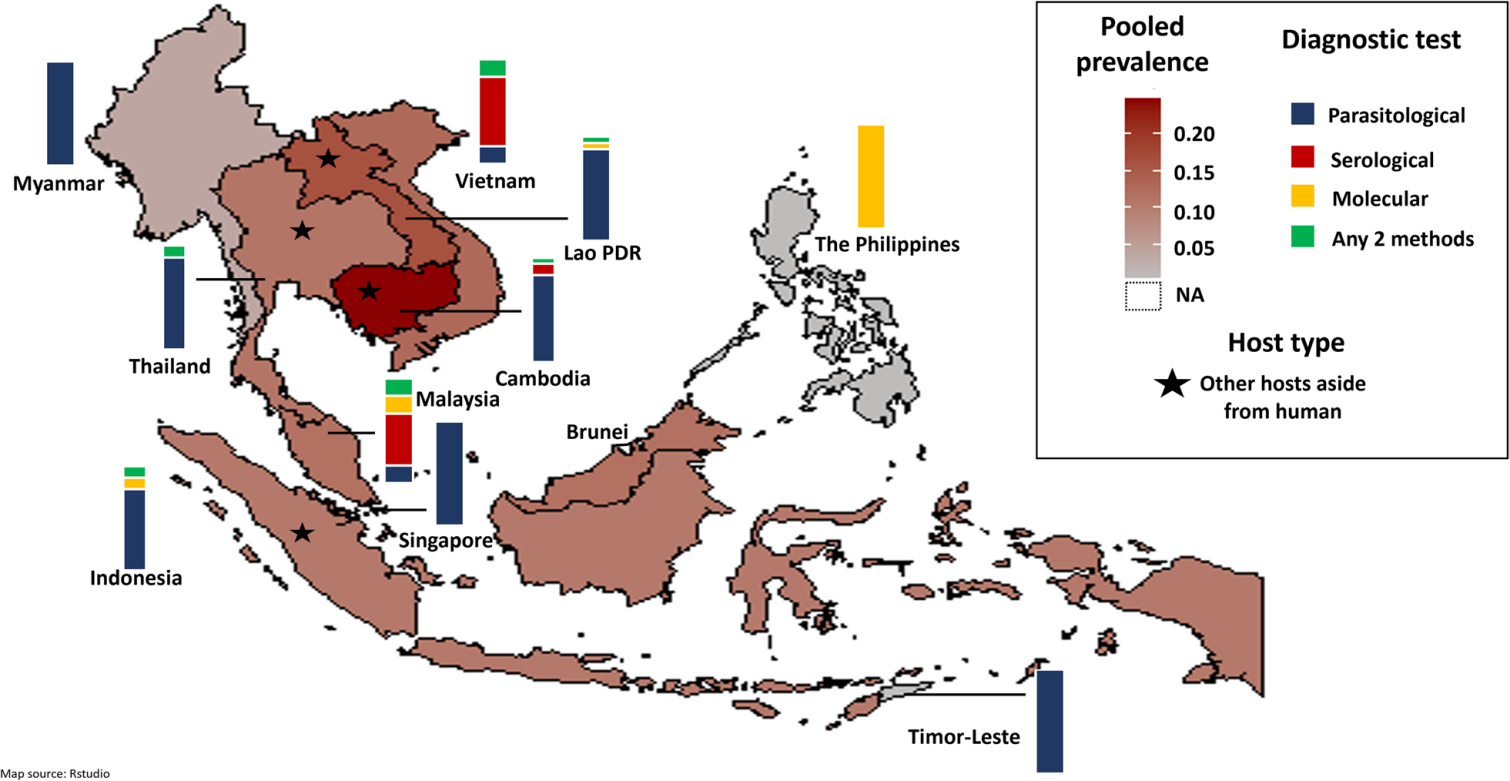
The pooled prevalence of *S. stercoralis* in Southeast Asia. The estimated pooled prevalence obtained for the 11 countries is indicated in the graduated shading (0 to 100%). The stacked bar chart indicates the proportion of diagnostic techniques (Blue: parasitological, red: serological, yellow: molecular, green: a combination of any two of the above categories) used for each country. The star indicates studies reporting other host types of *S. stercoralis* besides humans

Among the 163 studies (excluding the IWV group) originating from the 10 countries in Southeast Asia, the overall pooled prevalence of *S. stercoralis* based on the random effects model was 12.7% (95% *CI* 10.70–14.80%). Based on the two time periods, the pooled prevalence of *S. stercoralis* before the millennium was 13.8% (95% *CI* 7.79–21.18%) and 13.1% (95% *CI* 11.08–15.30%) after the millennium. Subgroup analysis by country revealed that the prevalence rates ranged from 0.4 to 24.9%. Cambodia had the highest pooled prevalence (24.9%), followed by Lao PDR (16.5%). Timor-Leste, The Philippines, and Singapore each had only one study, so their prevalence data were not pooled and instead based on the single study obtained for each. Figure 2 presents the estimated prevalence of *S. stercoralis* in Southeast Asia, along with the proportion of diagnostic test types used per country. Parasitological tests were the preferred diagnostic method in 58% of studies.

Moreover, parasitological techniques were used as the primary diagnostic test in seven (Myanmar, Lao PDR, Thailand, Cambodia, Indonesia, Timor-Leste, and Singapore) of the 10 countries. Aside from humans, *S. stercoralis* was reported in other host types, including dogs, cats, rodents, and transport carriers such as cockroaches and vegetables from markets. The four countries that had studies from other hosts aside from humans are Thailand, Cambodia, Vietnam, and Indonesia.

#### Thailand

**Fig. 3:**
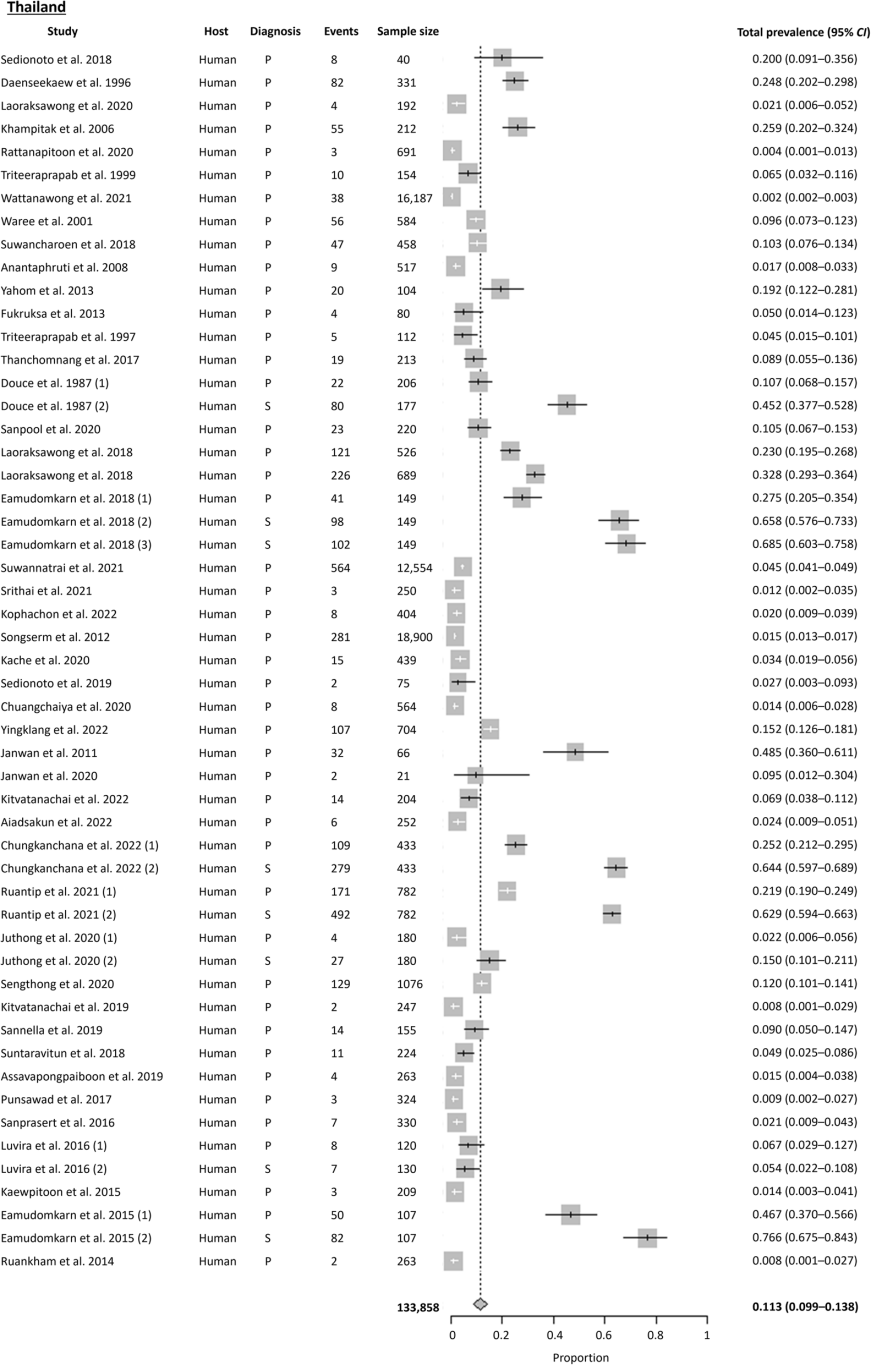
Forest plot of *S. stercoralis* prevalence in Thailand (1). The diagnostic techniques are abbreviated as P: Parasitological; S: Serological; M: Molecular; NA: Not available

**Fig. 4:**
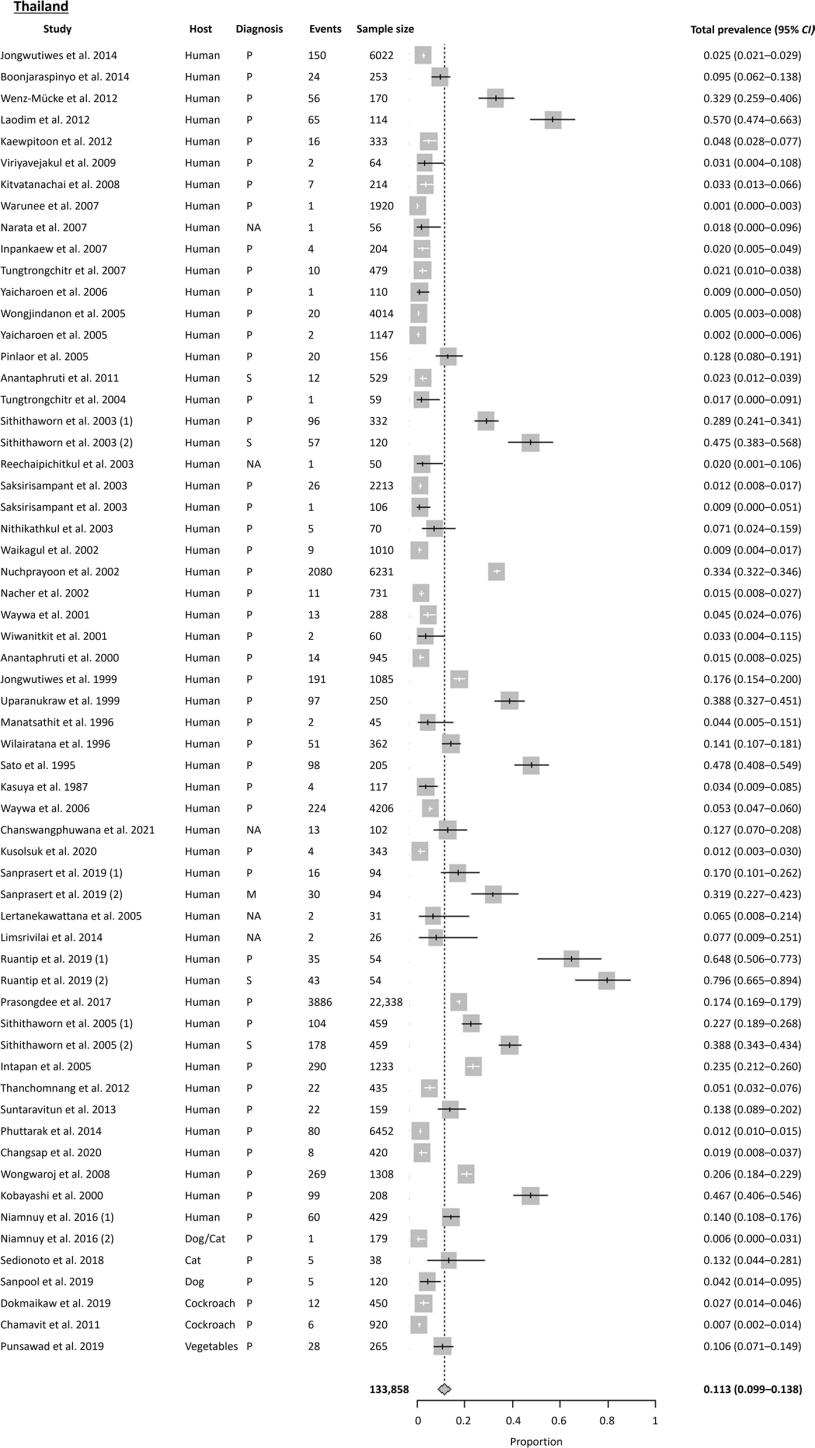
Forest plot of *S. stercoralis* prevalence in Thailand (2). The diagnostic techniques are abbreviated as P: Parasitological; S: Serological; M: Molecular; NA: Not available

A total of 98 publications were available from Thailand, with most studies conducted in the Northeast region. The Cochran Q test (*P* < 0.0001) and *I*^2^ index indicated a high level of heterogeneity (99.4%) among the studies. Publication bias was also detected using Egger’s test (*t* = 4.118, *P* = 0.015) and Begg’s test (*P* = 0.008). Based on the random effects model, the estimated country-wide pooled prevalence of *S. stercoralis* was 11.3% (95% *CI* 8.98–13.85%), slightly lower than the overall pooled prevalence of Southeast Asia (as shown in Figs. 3 and 4). Further analysis based on the four regions (Central, North, Northeast, and South) revealed that the highest prevalence of *S. stercoralis* was observed in the Northeast at 22.5%, followed by the North (15%), South (8.3%), and Central (6.6%) regions. Additionally, the results based on the two time periods showed a decreasing trend of *S. stercoralis* prevalence in the last two decades. The pooled prevalence obtained were was 17.7% (95% *CI* 9.19–28.13%) before the millennium and 11.3% (95% *CI* 8.83–13.93%) after the millennium.

Not only were human hosts affected, but domestic dogs, cats, and various transport carriers such as cockroaches and vegetables from markets were also found to habor *S. stercoralis*. Of the three studies focusing on domestic animals, *S. stercoralis* were also present in dogs and cats along with their owners who tested positive [19–21]. Regarding diagnostic tests, parasitological methods were the most commonly used (88.4%), with *S. stercoralis* larvae primarily detected using the agar plate culture (APC) or the formalin-ether concentration technique (FECT). Serological methods were usually employed alongside parasitological methods, with higher prevalence obtained using serology as compared to parasitological methods [22]. Aside from serum-based ELISA assays, an urine-based ELISA IgG was also employed [22, 23]. However, the limitations of serology was observed in immunocompromised patients, where Luvira et al. 2016 reported a prevalence of 5.4% in immunocompromised patients, with 43% sensitivity and 96% specificity [24].

#### Cambodia

**Fig. 5:**
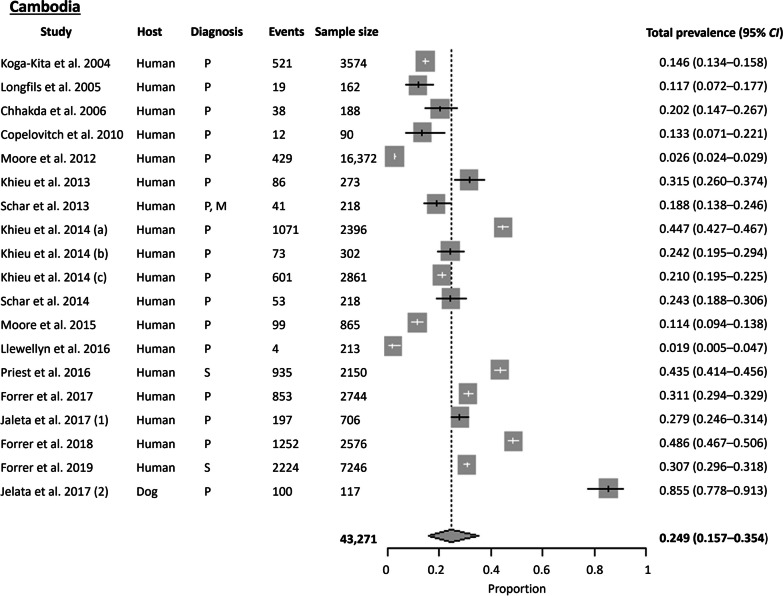
Forest plot of *S. stercoralis* prevalence in Cambodia. The diagnostic techniques are abbreviated as P: Parasitological; S: Serological; M: Molecular; NA: Not available

A total of 18 records from Cambodia were available, with most studies conducted in Preah Vihear Province in Northwestern Cambodia. The Cochran Q test (*P* < 0.0001) and *I*^2^ index revealed a high level of heterogeneity (99.8%) among the studies. No publication bias was observed using Egger’s test (*t* = 11.760, *P* = 0.096) and Begg’s test (*P* = 0.263). Among the 10 Southeast Asia countries compared in this study, Cambodia had the highest pooled prevalence at 24.9% (95% *CI* 15.65–35.38%) using the random effects model (Fig. 5). Subgroup analysis showed that the highest prevalence of *S. stercoralis* was in the Elephant Mountains and Cardamon region (33.7%), followed by the Northwestern (31.4%), Eastern (27.75%), and Mekong lowland (23.09%) regions.

A country-wide study across all 25 provinces using urine-based ELISA IgG serology found a prevalence of 30.7% [25]. *Strongyloides stercoralis* was ubiquitous and distributed throughout the country, with the highest prevalence in Koh Kong Province, followed by Kampong Speu Province (both provinces are in the Elephant Mountains and Cardamon region). The study also indicated that the risk of *S. stercoralis* infection increases with age. Also, using multiplex bead antibody serological assays, Priest et al. 2016 surveyed women of child-bearing age across 21 provinces in Cambodia and reported a prevalence of 45.9% [26]. Apart from humans, dogs were also found to be positive for *S. stercoralis* infection. Jelata et al. 2017 tested both humans and dogs from the same household and discovered a high *S. stercoralis* prevalence of 85% in dogs using parasitological techniques such as Baermann, APC, and Kato-Katz. Meanwhile, the prevalence in humans was 27% [27].

Parasitological methods were the most common means of detecting *S. stercoralis* (83.9%), with studies typically employing a combination of Kato-Katz, Baermann, and APC. Two studies used serology to survey on a national scale survey [25, 26].

#### Lao People’s Democratic Republic

**Fig. 6:**
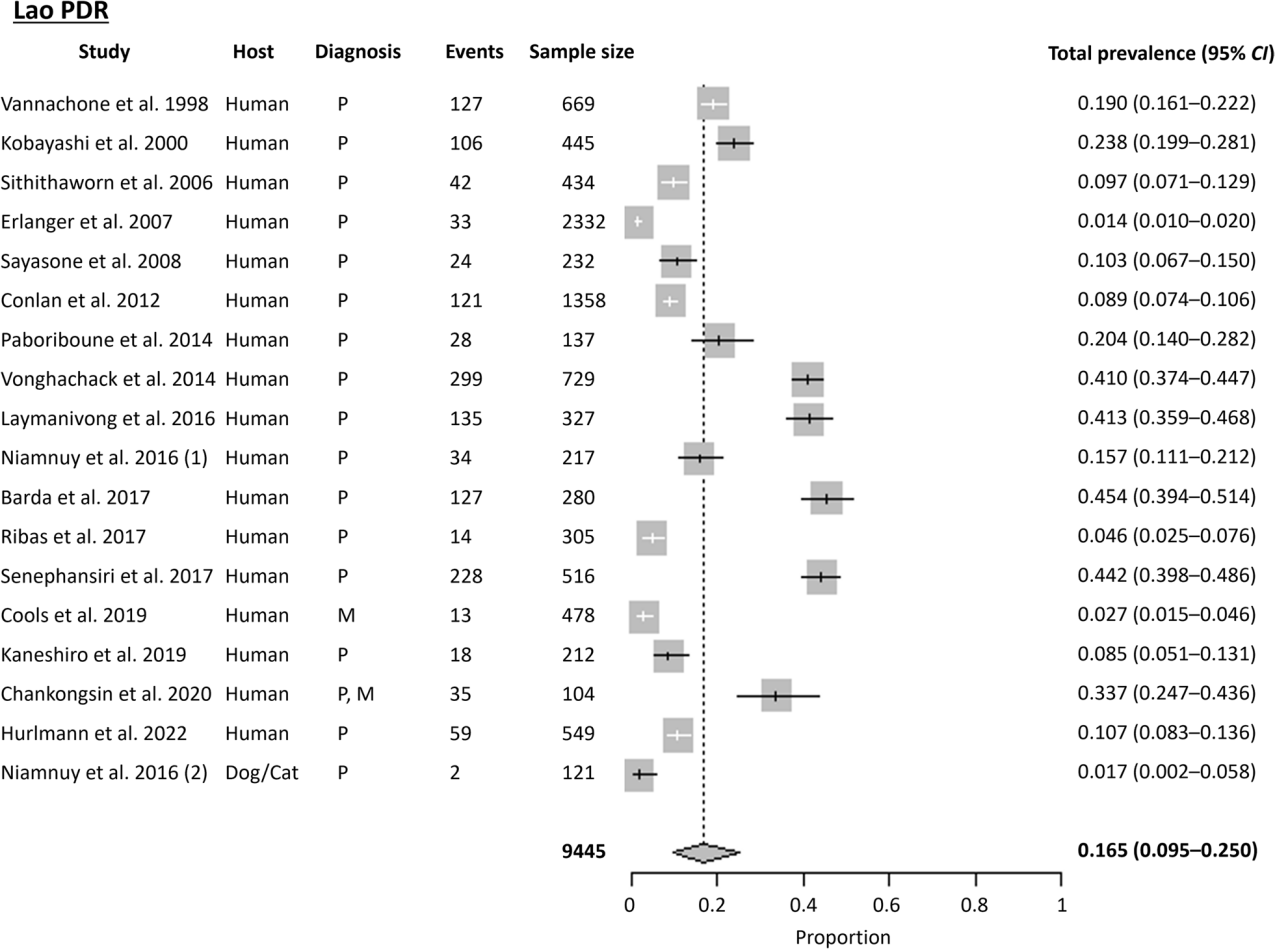
Forest plot of *S. stercoralis* prevalence in Lao PDR. The diagnostic techniques are abbreviated as P: Parasitological; S: Serological; M: Molecular; NA: Not available

Seventeen records on the prevalence of *S. stercoralis* were available for Lao PDR, with studies conducted in 11 out of the 17 provinces. The Cochran Q test (*P* < 0.0001) and *I*^2^ index indicated a high level of heterogeneity (99.0%) among the studies. No publication bias was observed using Egger’s test (*t* = 9.095, *P* = 0.096) and Begg’s test (*P* = 0.916). The pooled prevalence of *S. stercoralis* using the random effects model was 16.5% (95% *CI* 9.50–24.95%), making Lao PDR the country with the second-highest pooled prevalence of *S. stercoralis* after Cambodia (Fig. 6). The South region had the highest prevalence at 28%, followed by the North and Central regions at 17.9% and 14.8%, respectively.

Of the 17 studies, the highest prevalence (45%) of *S. stercoralis* was detected in residents in Champasak Province, where the Baermann method was used to identify *S. stercoralis* larvae in fecal samples [28]. Co-infections of *S. stercoralis* with other soil-transmitted helminths such as hookworm and *Trichuris trichiura* were also present in the studies from Lao PDR [29–31]. Two studies focused on HIV-infected patients, where the prevalence of *S. stercoralis* was 8.5% using direct microscopy and 20.4% using a combination of direct, Kato-Katz, and concentration techniques [29, 32]. Similar to findings in Thailand and Cambodia, *S. stercoralis* was also detected in domestic dogs or cats, with Niamnuy et al. 2016 reporting a prevalence of 1.7% in domestic animals and 15.7% in their owners [21].

Parasitological techniques were the diagnostic method of choice in 15 studies, with none using serological methods. A comparison of parasitological and molecular techniques for *S. stercoralis* detection showed that combining both techniques yielded the best results. By employing a combination of Baermann, APC, and PCR, 33.7% tested positive for *S. stercoralis*, while using only Baermann and APC or PCR detected 20.2% and 25%, respectively [33].

#### Malaysia

**Fig. 7:**
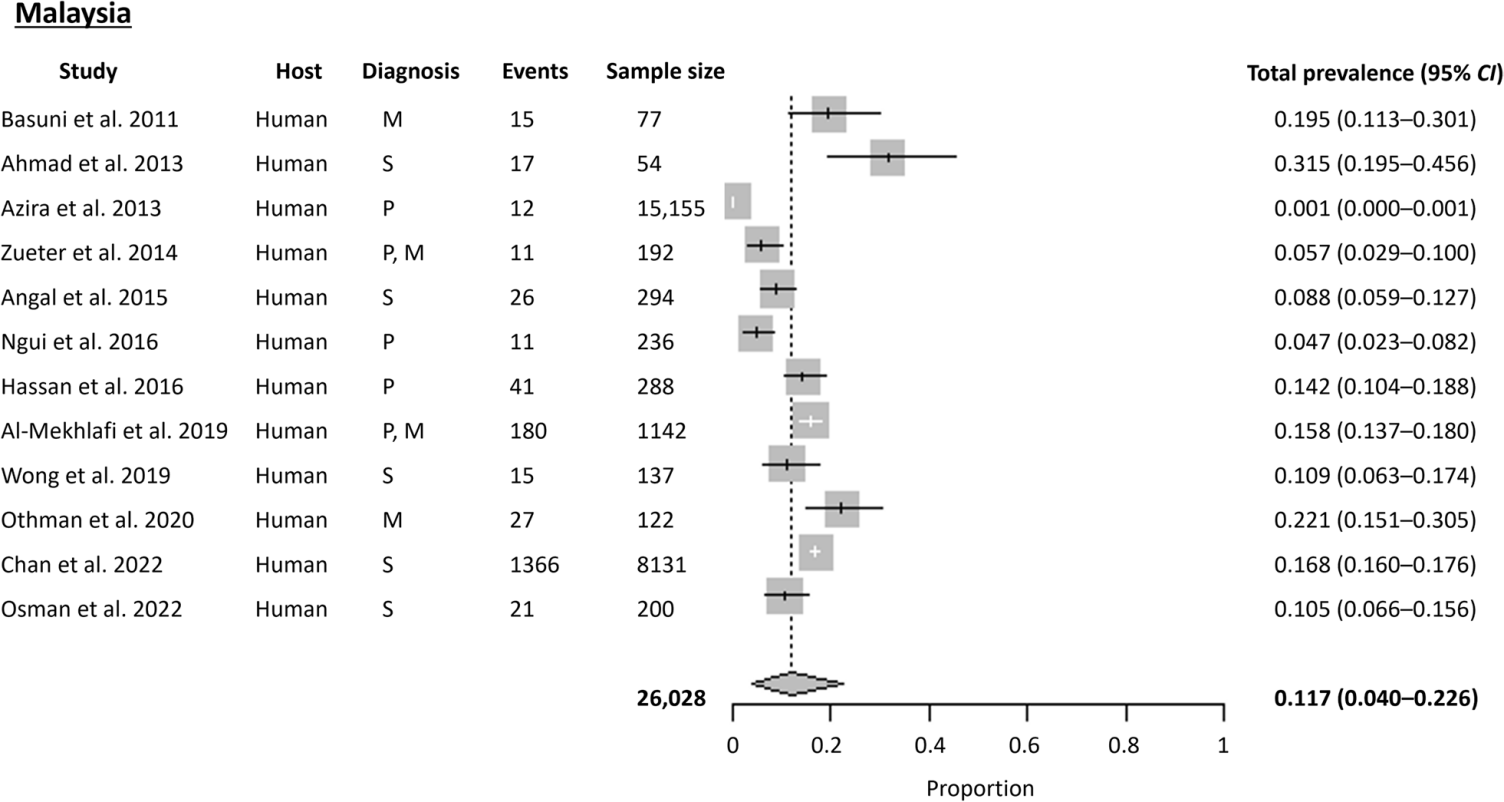
Forest plot of *S. stercoralis* prevalence in Malaysia. The diagnostic techniques are abbreviated as P: Parasitological; S: Serological; M: Molecular; NA: Not available

A total of 12 articles were available for Malaysia, covering both Peninsular and East Malaysia. Nine articles were from Peninsular Malaysia, while three were from East Malaysia. The Cochran Q test (*P* < 0.0001) and *I*^2^ index indicated a high level of heterogeneity (99.7%) among the studies. No publication bias was observed using Egger’s test (*t* = 8.623, *P* = 0.186) and Begg’s test (*P* = 0.956). The estimated country-wide pooled prevalence for *S. stercoralis* was 11.7% (95% *CI* 3.99–22.58%), slightly lower than that of Southeast Asia collectively (Fig. 7). Comparing the prevalence between the two regions, East Malaysia (20.7%) had a higher prevalence than Peninsular Malaysia (13.2%).

All 12 studies focused on human hosts, with the highest prevalence of *S. stercoralis* detected among the Orang Asli communities in the State of Selangor at 31.5%, using ELISA IgG serology [34]. Moreover, 76.5% of the *S. stercoralis* seropositive participants were positive for other helminths detected through microscopy. Surveying Orang Asli communities positive for tuberculosis across six states in Malaysia, Wong et al. 2019 revealed a seroprevalence of *S. stercoralis* was 10.9% [35]. Two more studies on Orang Asli communities reported prevalences of 22.1% and 15.8% [36, 37]. Comparing the prevalence of *S. stercoralis* among HIV-negative and HIV-positive prison inmates, Angal et al. 2015 found a higher prevalence among HIV-negative inmates, contrasting with other studies [38].

The studies from Malaysia employed the most diverse range of diagnostic methods, with 50% using serology and the remaining 50% using parasitological, molecular, or a combination of any two methods. Additionally, higher sensitivity of molecular methods compared to parasitological methods (without larva culture) was demonstrated, with molecular methods detecting 19.4% while Kato-Katz, FECT, and direct smear detecting only 2.5% [39]. Similarly, Othman et al. 2020 showed that *S. stercoralis* was not detected using FECT and direct smear, while PCR results were able to detect 22.1% [37].

#### Indonesia

**Fig. 8:**
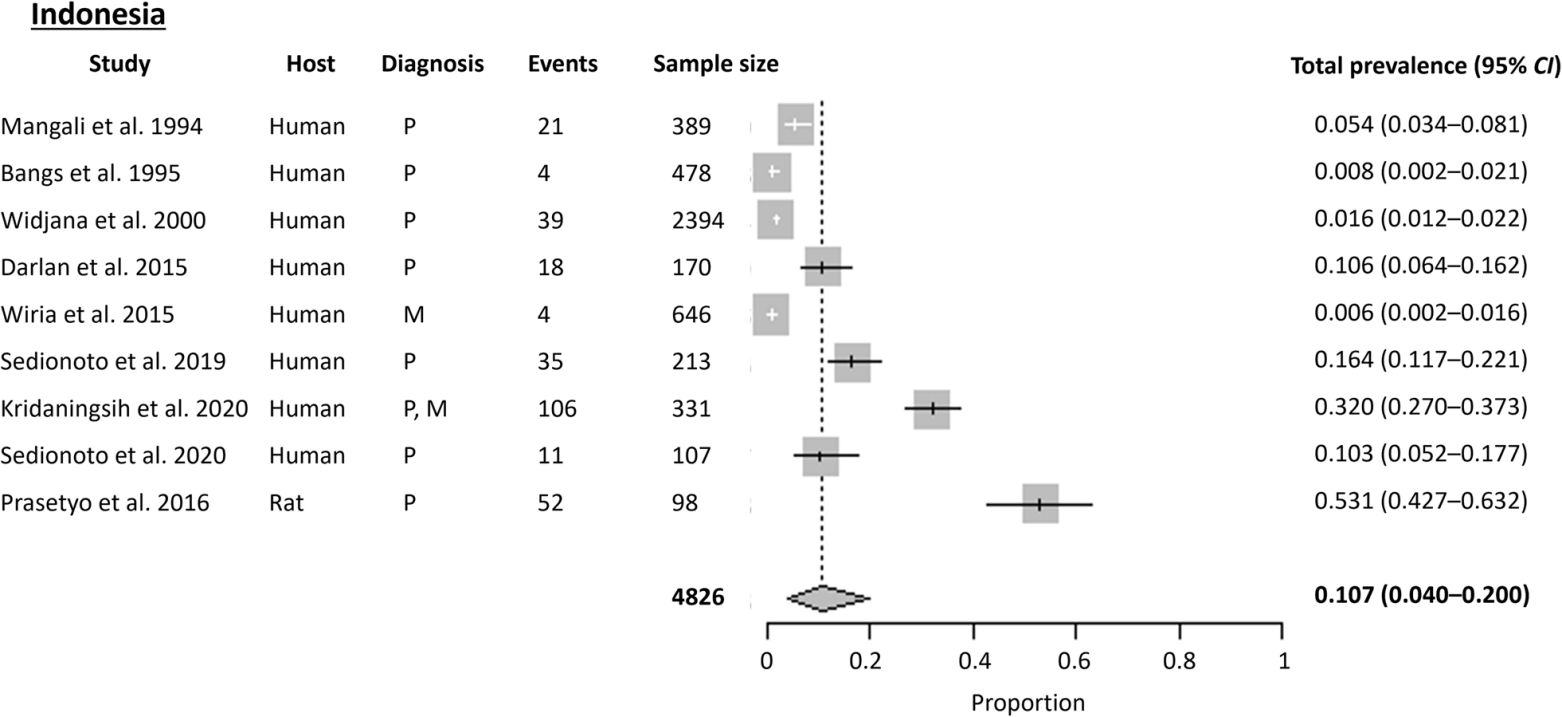
Forest plot of *S. stercoralis* prevalence in Indonesia. The diagnostic techniques are abbreviated as P: Parasitological; S: Serological; M: Molecular; NA: Not available

A total of nine articles were obtained from Indonesia, with studies conducted in only six out of the 38 provinces. The Cochran Q test (*P* < 0.0001) and *I*^2^ index revealed a high level of heterogeneity (98.5%) among the studies. No publication bias was observed using Egger’s test (*t* = 9.038, *P* = 0.060) and Begg’s test (*P* = 0.089). The estimated pooled prevalence of *S. stercoralis* in Indonesia was 10.7% (95% *CI* 4.00–19.97%) using the random effects model (Fig. 8).

The highest prevalence of *S. stercoralis* was obtained from rodents in Surabaya Province, with a prevalence of 53%, while the highest prevalence in humans was in Papua Province [40, 41]. Moreover, 73.6% of *S. stercoralis*-infected participants were co-infected with other soil-transmitted helminths.

Of the nine articles, six (77.8%) used parasitological methods, usually utilizing a combination of Kato-Katz with APC or Harada-Mori culture, for diagnosis. The low sensitivity of Kato-Katz was demonstrated again, as *S. stercoralis* was not detected using Kato-Katz, while using APC yielded a prevalence of 16.4% [42].

#### Vietnam, Myanmar, Philippines, Timor-Leste, Singapore

Eleven articles were obtained from these five countries, with one study each from Timor-Leste, The Philippines, and Singapore (Additional file 2). Based on one study each, the prevalence of *S. stercoralis* in Timor-Leste, The Philippines, and Singapore were 0.4%, 0.8%, and 3.7%, respectively. However, as the study from Singapore was conducted in the 1970s, the actual prevalence might not reflect the current situation.

For Myanmar, two studies were available, with a pooled prevalence of 3.9% (95% *CI* 0.77–9.06%). They were conducted in Yangon, Ayeyarwady, and Bago-East divisions, and both studies used parasitological methods (APC and Kato-katz) for detection. For Vietnam, six studies were available, with a pooled prevalence of 13% (95% *CI* 5.23–23.56%). The highest prevalence of strongyloidiasis was reported in Hanoi Medical Center in Hanoi city in 2018, with a prevalence of 46.3% among visitors from 27 northern provinces using ELISA IgG [43]. Another study, conducted between 2016 and 2017 and surveying patients from the same 27 provinces, found a seroprevalence of 20%, indicating an increase in the prevalence of the disease [44].

#### Immigrants, workers, veterans

**Fig. 9:**
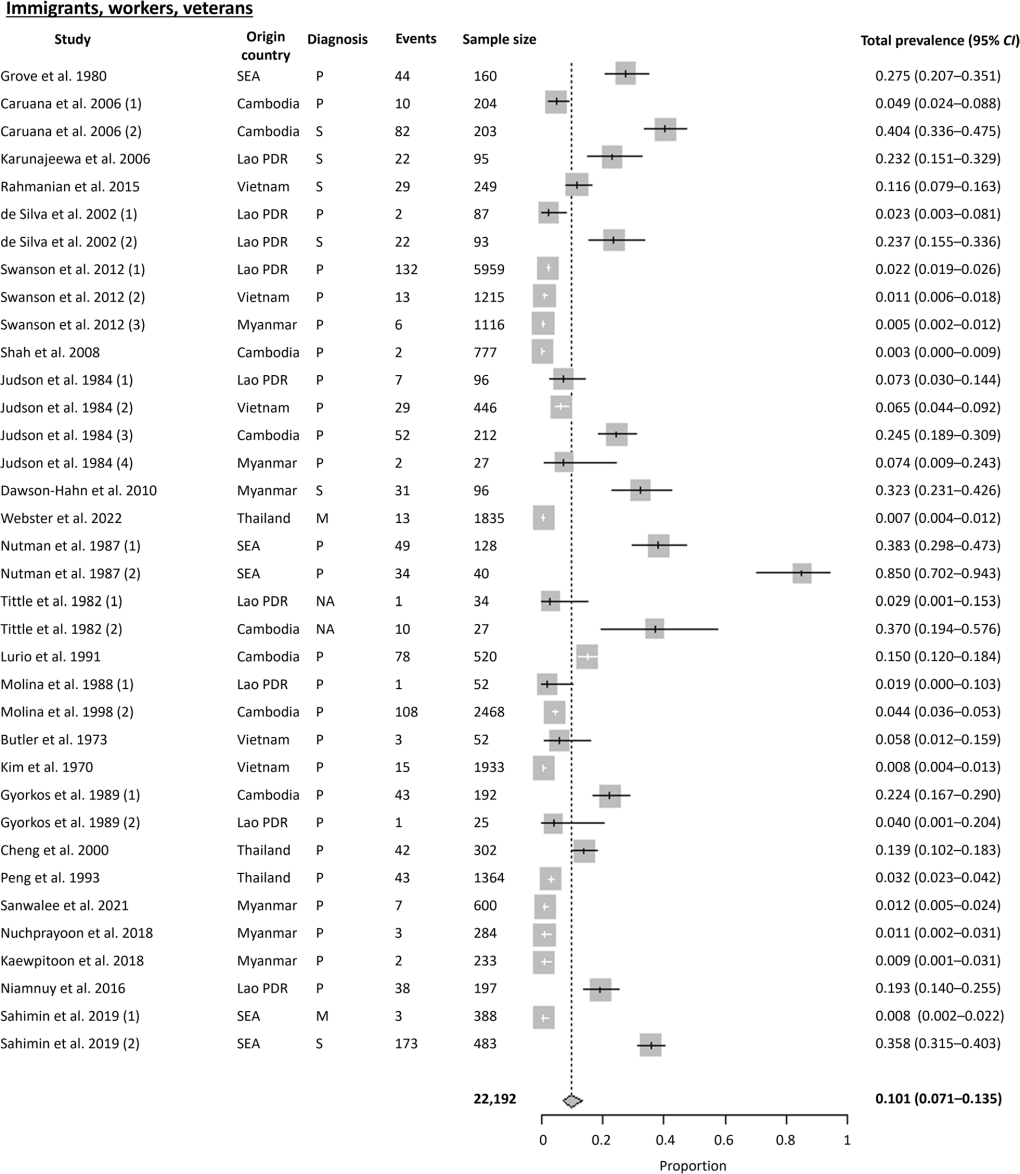
Forest plot of *S. stercoralis* prevalence among immigrants, workers, and veterans from Southeast Asia. The diagnostic techniques are abbreviated as P: Parasitological; S: Serological; M: Molecular; NA: Not available

Among the studies analyzed, a notable proportion (12.6%) focused on individuals belonging to the following categories: immigrants, workers, and veterans. These groups encompass: (1) immigrants from Southeast Asia who have resettled in other countries, (2) migrant workers from Southeast Asia who are currently employed in other countries, and (3) veteran soldiers who previously served in the war in Southeast Asia. The Cochran Q test (*P* < 0.0001) and *I*^2^ index revealed a high level of heterogeneity (97.9%) among the studies. No publication bias was observed using Egger’s test (*t* = 6.818, *P* = 0.0003) and Begg’s test (*P* = 0.02). A pooled prevalence of 10% (95% *CI* 7.06–13.52%) was obtained, with people originating from six countries in Southeast Asia (Cambodia, Lao PDR, Vietnam, Myanmar, Thailand, and Indonesia) (Fig. 9). The countries where they reside or work include Australia, The United States of America (USA), Canada, Thailand, and Malaysia.

The highest *S. stercoralis* prevalence was from a study by Nutman et al. 1987, with a seroprevalence of 85% among new immigrants to USA from the Indochina region (Cambodia, Lao PDR and Vietnam) were reported in 1987 [45]. In 2006, a seroprevalence of 40% was detected in Cambodian communities that resettled in Australia [46]. Among the studies conducted on veteran soldiers, a seroprevalence of 11.6% was detected among Australians involved in war operations in Vietnam [47]. For migrant workers, a prevalence of 19.3% was obtained using FECT and Harada-mori culture from Laotian workers working in Thailand (Ubon Ratchathani Province) [21]. A similar high prevalence of 13.9% also resulted from using FECT from Thai workers working in Taiwan, China [48].

#### *Strongyloides* species in Southeast Asia

Aside from *S. stercoralis*, four other species of *Strongyloides* were present in Southeast Asia. These four species are *S. fuelleborni*, *S. ratti*, *S. papillosus*, and *S. ransomi*. Additional file 3 presents the other *Strongyloides* species found in Southeast Asia, with their respective host, country, and prevalence.

Seven studies reported *S. fuelleborni* originating from Lao PDR, Thailand, and Malaysia. Their hosts include humans and nonhuman primates such as long-tailed macaques, pig-tailed macaques, white-handed gibbons, and Bornean orangutans. Janwan et al. 2020 found *S. fuelleborni* in pig-tailed macaques and their owners [49]. The prevalence of *S. fuelleborni* in pig-tailed macaques was 51.1%, while *S. fuelleborni* was detected in one owner. Moreover, Thanchomnang et al. 2017 detected both *S. stercoralis* and *S. fuelleborni* in humans living near Kumphawapi Monkey Park in Udon Thani Province [8]. A high prevalence of *S. fuelleborni* found in nonhuman primates (ranging from 31.1 to 62.1%), along with the evidence of infection in human hosts, demonstrates the potential of the zoonotic transmissibility of *S. fuelleborni* to humans.

## Discussion

This systematic review and meta-analysis provided a comprehensive overview of the prevalence of *Strongyloides stercoralis* and other *Strongyloides* species in Southeast Asia. Our findings demonstrate considerable heterogeneity in prevalence rates among countries in the region, with variations in diagnostic methods used across studies. These results underscore the importance of continued research and surveillance efforts to understand better and control *Strongyloides* infections in Southeast Asia.

The overall pooled prevalence of *S. stercoralis* from this systematic review and meta-analysis was estimated at 12.7% in Southeast Asia, based on the data obtained from 187 publications across 10 countries. Additionally, the results based on the division into two time periods (before and after the millennium) showed that the prevalence of *S. stercoralis* did not change significantly in the region over time. We also revealed that *S. stercoralis* in Southeast Asia ranged from 0.4 to 24.9%, with the highest prevalence in Cambodia. A recent estimate conducted by Buonfrate et al. 2020 on the global prevalence of *S. stercoralis* through modeling was 8.1%, with a 10–15% prevalence estimated for Southeast Asia [1]. However, the prevalence of *S. stercoralis* could still be underestimated. One possible reason is that most studies used parasitological methods, such as direct microscopy, Kato-Katz, and FECT, instead of parasitological methods involving larva culture as they are more convenient and less time-consuming [50, 51]. Another reason for the underestimation is the skewed proportion of studies originating from some countries in Southeast Asia. Our results demonstrated that more than 50% of the studies were from Thailand, while the other 50% was shared between the nine other countries in Southeast Asia. For example, many regions were not covered in Indonesia, where studies were conducted in six out of 38 provinces, and in The Philippines, where only one was conducted in Laguna Province.

Of the countries in Southeast Asia, the two countries with the highest pooled prevalence of *S. stercoralis* was Cambodia (24.9%) and Lao PDR (16.5%). Also, most of the studies in these two countries were conducted after the millennium. This could be a reason why the overall prevalence of *S. stercoralis* in Southeast Asia did not decrease in spite of the general improvements in santitiaton and hygiene in other countries regionally, whereas a decrease in *S. stercoralis* prevalence was observed in Thailand [52]. The high prevalence of *S. stercoralis* in Cambodia can be attributed to various factors such as climate, sanitation and hygiene, socio-economic conditions, occupation demographics, and the presence of domestic and free-roaming dogs [53]. Eslahi et al. 2022 conducted a meta-analysis of the global prevalence of *S. stercoralis* in dogs and found that the highest prevalence was in Cambodia, with more than 8% pooled prevalence [54]. Moreover, the genetic analysis also revealed a close relationship between type A (including humans, dogs, and other hosts) and type B (dog only) *S. stercoralis* genotypes [20, 55]. These two genotypes have been documented in Cambodia and Myanmar [27, 56]. Although the transmissibility and infectivity of the Type B *S. stercoralis* genotype to humans from dogs are currently not known, the risk of transmission is still plausible and a multi-faceted one-health approach for *S. stercoralis* control may be useful.

In Thailand, although the overall prevalence was 11.3%, there was a decrease in the prevalence of *S. stercoralis* before and after the millennium. Since the 2000s, emphasis was placed by the government to reduce helminthic infections in the country. The Thai Department of Disease Control has adopted an active control in high-risk areas (areas with parasite prevalence > 10%) through guidelines for endemic areas [57, 58]. These guidelines include screening, identification, treatment, and follow-up protocols. Additionally, through a Health Education and Preventive Equipment Package (HEPEP), health education, mass screening, and treatment for strongyloidiasis was successfully implemented in the Northeast region [59]. The decrease in strongyloidiasis after the millennium was also evidenced in a retrospective study performed using patient data from 2004 to 2014 [60]. The number of strongyloidiasis patients decreased from 22.4% to 2004 to 12.9% in 2014. The improvements in sanitation and hygiene, along with successful government policies and interventions could have led to the overall decrease in *S. stercoralis* prevalence in Thailand.

Although improvements have been made in Southeast Asia, *S. stercoralis* presence in the environment is still prevalent. Aside from being present in the soil and fecal material, *S. stercoralis* transmission can be facilitated by transport carriers such as cockroaches and vegetables [61–63]. Larvae were found in samples of various *Periplanta* species from three fresh markets in Chachoengsao Province and 18 open-air shopping markets in Samut Prakan Province, with 2.6% and 0.8% prevalence rates, respectively [61, 62]. Also, Punsawad et al. 2013 reported the presence of *S. stercoralis* larva on fresh vegetables obtained from open-air markets in Nakhon Si Thammarat Province [63]. Other possible factors driving the risk of transmission include global connectivity leading to increased people movement between and within countries and climate changes. First, the impact of global connectivity on the transmission risk of strongyloidiasis was observed with a significant proportion of studies in the IWV group having a pooled prevalence of 10%. With barriers reduced due to global connectivity and the ease and affordability of travel, strongyloidiasis is not only limited to tropical regions. Angheben et al. 2011 described two cases of strongyloidiasis in Italian tourists after traveling in Malaysia, Singapore, and Thailand [64]. A Swiss traveler also reported strongyloidiasis after visiting Vietnam and Cambodia [65]. With geographical barriers reduced, the classification of strongyloidiasis as a tropical and subtropical disease can be misleading. Second, although the favorable conditions for transmission of *S. stercoralis* are warm and moist soil, studies have demonstrated that the larva can survive lower temperatures [66]. The increasing global average temperatures may also increase the chances of the larva’s survival in temperate countries. Beknazarova et al. 2016 explored the global prevalence of strongyloidiasis and provided evidence to show that infection is primarily determined by the socio-economic status of communities rather than by geography or climate [67]. With an increase in global connectivity coupled with climate changes, the importance of screening programs and accurate diagnosis for strongyloidiasis is warranted.

Currently, there is no standard requirement for the diagnosis of strongyloidiasis. Although larva culture along with serology is recommended by the WHO, no stringent protocols are in place for screening [17]. Also, molecular tests should be clinically validated, especially in large-scale field-based settings [17]. Molecular identification of *Strongyloides* is also essential to accurately assess the risk of transmission and their zoonotic potential since both animals and humans can be infected with the same *Strongyloides* species.

For sustainable control of *Strongyloides* infections, a multifaceted approach is necessary. Strategies include improving sanitation and hygiene practices and implementing health education programs to raise awareness about the risks and associated preventive measures. Integrating *Strongyloides* screening into routine health services for vulnerable populations should also be considered. In addition, strengthening surveillance systems and fostering regional collaborations can facilitate sharing of best practices and resources to address the challenges associated with *Strongyloides* control.

## Conclusions

This systematic review and meta-analysis highlight the importance of the region’s ongoing research, surveillance, and control efforts. The use of inadequate diagnostic methods remains a significant challenge, resulting in low detection sensitivity and the underestimation of disease burden. Additionally, the impact of global connectivity and climate changes on transmission necessitates a multi-pronged approach for effective intervention and control. To address strongyloidiasis in Southeast Asia, we recommend (1) widespread adoption of appropriate diagnostic tests, (2) screening programs especially those requiring immunosuppressive drug treatments and travelers, (3) mass drug administration for humans [68], and (4) integration across various sectors for a One Health approach to strongyloidiasis control. Progress in these areas at the regional level could significantly contribute to sustainable control of strongyloidiasis worldwide.

### Supplementary Information

**Additional file 1.** PRISMA checklist.

**Additional file 2.** *Strongyloides stercoralis* data extracted from articles.

**Additional file 3.** Other *Strongyloides* species data extracted from articles.

## Data Availability

All data generated or analyzed during this study are included in this published article and its additional information files.

## Abbreviations

APC: Agar plate culture
ELISA: Enzyme-linked-immunosorbant assay
FECT: Formalin-ether concentration technique
HIV: Human immunodeficiency virus
Lao PDR: Lao People’s Democratic Republic
PCR: Polymerase chain reaction
STH: Soil-transmitted helminth
WHO: World Health Organization
USA: The United States of America
